# Synthesis and Characterization of Phenazine-Based Redox Center for High-Performance Polymer Poly(aryl ether sulfone)-5,10-Diphenyl-dihydrophenazine

**DOI:** 10.3390/molecules29071618

**Published:** 2024-04-03

**Authors:** Qilin Wang, Xuehan Wang, Yuehui Zhai, Zhibo Zheng, Huilin Shen, Yuntao Han, Zheng Chen, Zhenhua Jiang

**Affiliations:** 1 Engineering Research Center of Special Engineering Plastics, Ministry of Education, National and Local Joint Engineering Laboratory for Synthetic Technology of High Performance Polymer, College of Chemistry, Jilin University, Qianjin Street 2699, Changchun 130012, China; wangql22@mails.jlu.edu.cn (Q.W.); xuehan23@mails.jlu.edu.cn (X.W.); shenhl22@mails.jlu.edu.cn (H.S.); jiangzhenhua@jlu.edu.cn (Z.J.); 2 School of Materials and Energy, University of Electronic Science and Technology of China, Chengdu 611731, China; zhaiyuehui@uestc.edu.cn; 3 Department of Chemical Engineering and Applied Chemistry, College of Chemistry, Jilin University, Qianjin Street 2699, Changchun 130012, China; zhengzb22@mails.jlu.edu.cn

**Keywords:** poly(aryl ether sulfone), phenazine, electrochromism, organic battery cathode

## Abstract

Phenazine-based redox-active centers are capable of averting chemical bond rearrangements by coupling during the reaction process, leading to enhanced stabilization of the material. When introduced into a high-performance polymer with excellent physicochemical properties, they can be endowed with electrochemical properties and related prospective applications while maintaining the capabilities of the materials. In this study, a facile C-N coupling method was chosen for the synthesis of serial poly(aryl ether sulfone) materials containing phenazine-based redox-active centers and to explore their electrochemical properties. As expected, the cyclic voltammetry curves of PAS-DPPZ-60, which basically overlap after thousands of cycles, indicate the stability of the electrochemical properties. As an electrochromic material, the transmittance change in PAS-DPPZ-60 exhibits only a slight attenuation after as long as 600 cycles. Meanwhile, as an organic battery cathode material, PAS-DPPZ has a theoretical specific capacity of 126 mAh g^−1^, and the capacity retention rate is 82.6% after 100 cycles at a 0.1 C current density. The perfect combination of advantageous features between phenazine and poly(aryl ether sulfone) is considered to be the reason for the favorable electrochemical performance of the material series.

## 1. Introduction

Polymers possess various advantages including light weight, corrosion resistance, and molding ease, and have thus gained popularity in all walks of life throughout the development of society. Nevertheless, pure polymers typically suffer from their own inherent defects including poor thermal resistance and limited mechanical strength, a problem that urgently needs to be solved [1,2,3,4,5,6]. The emergence of high-performance special engineering plastics has increased the adaptability of plastics in harsh conditions to a certain extent [7,8,9]. Poly(aryl ether sulfone) (PAS) is a class of high-performance polymer materials with comprehensive properties formed by the interpenetration bond of an ether bond and a sulfone group [10]. The aromatic ring structure in the backbone is rigid, which is found in large amounts, imparting to the resin exceptional levels of heat resistance, radiation resistance, creep resistance, and flame retardancy. The sulfone group in the molecular chain is a steric structure, which prevents the polymer from being melt-crystallized; thus the resin base exhibits an amorphous nature and a highly transparent state. The sulfur element contained in the sulfone group lies at the highest oxidation state, which enhances the oxidative stability of the polymer. The presence of ether bonds and sulfone groups makes the PAS molecular chain’s flexibility increase, providing great toughness and ductility for the resin, while the increased chain flexibility allows PAS to be suitable for a variety of processing and molding methods as well as improves its resistance to high-temperature hydrolysis. The superior overall performance of PAS enables it to have extensive applications in the military, machinery manufacturing, petrochemical, electronics, transport, medical equipment, and other high-tech industries [11,12,13,14,15]. The various structures of poly(aryl ether sulfone) are quite similar, all of which are prepared by a nucleophilic polycondensation reaction between monomers containing a diphenylsulfone moiety structure and corresponding biphenol monomers under certain conditions [16,17]. In other words, different functionalized poly(aryl ether sulfone) materials can be attained by selecting bisphenol monomers containing diverse functional units. The aromatic ring, ether bond, and sulfone group still form the primary structure of the functionalized polymer. Therefore, the functionalized PAS retains the excellent properties of poly(aryl ether sulfone) materials to a large extent. Moreover, there are varying functionalized structures that allow poly(aryl ether sulfone) materials to meet the needs of various applications with corresponding properties [18,19,20,21,22]. In view of employing the remarkable properties of PAS and endowing it with electrochemical activity so that it can satisfy the demands of relevant applications in the field of electrochemistry, it is obviously necessary to modify the PAS material itself.

As a classical aromatic nitrogen-containing heterocyclic compound, phenazine is non-biotoxic and synthesized from low-cost source materials. Benefiting from its remarkable properties regarding redox activity [23,24], photosensitivity, conductivity, and structural modifiability, phenazine and its derivatives have been extensively employed in the fields of batteries, fluorescent probes, dyes, conductive agents, magnetic materials, and photocatalysts [25,26,27,28,29]. More surprisingly, the effective positive charge dispersion in the conjugated structure enables the phenazine unit to be electrically active with multi-site reactions occurring in situ, with almost no change in the skeleton, which prevents chemical rearrangement. Once oxidized, the π electrons in the periphery of the phenazine transform from 4n to 4n+2, and the discrete dispersion of the large π-electron cloud formed by the electron loss in the conjugated system occurs, thereby stabilizing the charge center. Simultaneously, the aromaticity in the system turns from the initial weak anti-aromaticity to aromaticity. According to Hückel’s law, the corresponding phenazine cation becomes even more stable [30]. In recent years, researchers have linked several functional groups with specific properties to phenazine groups to obtain more characteristic functionalized phenazine derivatives [31,32,33,34,35]. Integrating the unique performance of phenazine with the virtues provided by the introduced moiety not only promotes the evolution of phenazine derivatives but also enriches their variety and applications among intelligent substances.

Herein, phenazine was selected as the electroactive center and poly(aryl ether sulfone) as the basic backbone to construct functionalized poly(aryl ether sulfone) materials via a facile and efficient C–N coupling method [36]. The synthesized serial polymers fulfilled the design strategy as expected by combining the excellent and steady electrochemical activity of phenazine with the prominent comprehensive performance of PAS itself. Phenazine-based functionalized PAS suitable for diverse alternative applications were fabricated with adjusted phenazine moiety occupancy ratios. Among them, PAS-DPPZ-60, employed as an electrochromic material, exhibited just a slight degradation in optical contrast (ΔT) following up to 12,000 s (600 cycles) of circulation. PAS-DPPZ, meanwhile, a candidate as an organic battery cathode material, demonstrated a theoretical specific capacity of 126 mAhg^−1^ and a promising battery performance, with a capacity maintained up to 82.6% after 100 cycles at a 0.1 C current density.

## 2. Results and Discussion

### 2.1. Monomer Synthesis

The 5,10-dihydrophenazine was prepared according to the published literature [37]. Briefly, 1.80 g phenazine (10 mmol) and 120 mL ethanol were added to a 250 mL three-necked flask and heated to steady reflux with nitrogen protection. Subsequently, 50 mL of a solution containing 3.48 g (20 mmol) of sodium dithionite was prepared in deionized water and slowly dripped into the reaction flask using a constant-pressure dropping funnel. The reaction continued for two hours. When the reaction solution was cooled to room temperature, it was filtered and washed three times each with deionized water and anhydrous ethanol. The obtained light green solid product was directly used in the following polymerization reaction after vacuum drying at 50 °C for 4 h.

### 2.2. Polymer Synthesis and Characterization

**Scheme 1:**
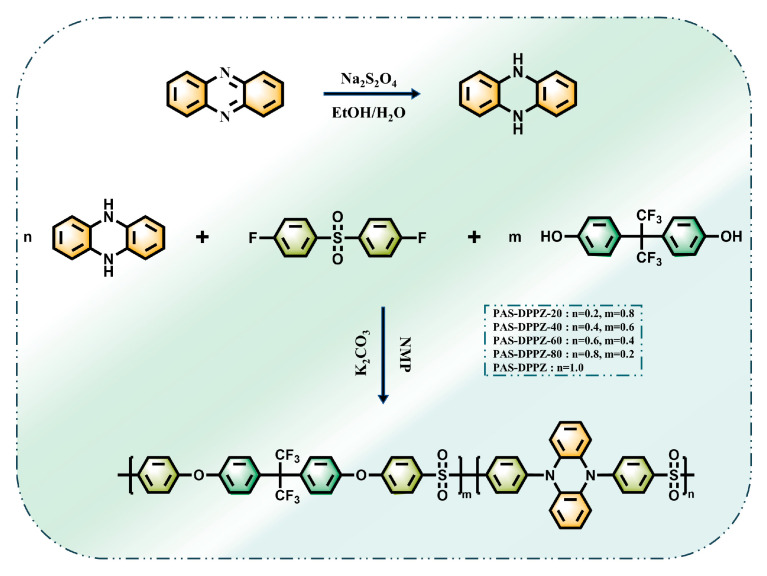
Synthesis routes of monomers and PAS-DPPZs.

Scheme 1 depicts the synthetic route of the target polymers in detail. Series of phenazine-based poly(aryl ether sulfone) polymers were prepared by a modified C–N coupling procedure [36]. The polymerization was carried out in N-methylpyrrolidone (NMP) under conditions of 2.5 equivalents K_2_CO_3_ as a catalyst through adapting the ratio of monomers 5,10-dihydrophenazine and bisphenol AF. The polymers obtained were washed with deionized water and anhydrous ethanol before purification by Soxhlet extraction to remove products with relatively low molecular weight. The polymeric materials with narrow molecular weight distribution were ultimately gained. Based on the proportion of phenazine-based monomer in the polymers, they were designated as PAS-DPPZ-20, PAS-DPPZ-40, PAS-DPPZ-60, PAS-DPPZ-80, and PAS-DPPZ. ^1^H NMR and FT-IR were selected for the structural characterizations of the subject polymers, and the results are shown in Figures S1 and S2. The FT-IR spectra of the polymers displayed duly characteristic absorption peaks near 1322 cm^−1^, 1303 cm^−1^, 1256 cm^−1^, and ^−^1109 cm^−1^ wavenumbers corresponding to the C-SO_2_-C, N(Ar)_3_, Ar-O-Ar, and CF_3_ stretching vibrations, as appropriate [38,39,40].

### 2.3. Solubility and Molecular Weight

The solubility properties determined how the polymers were processed in practical situations, and qualitative measurement results of polymer solubility are summarized in Table S1. At low proportions to phenazine-based monomers, the polymers had relatively favorable solubility in a wide range of conventional solvents including chloroform, tetrahydrofuran (THF), N,N-dimethylformamide (DMF), N,N-dimethylacetamide (DMAc), and so on. The dissolution behavior decreased as the percentage of phenazine-based monomers in the polymer increased. Once the percentage exceeded 60%, polymers were virtually insoluble in conventional solvents. The aforementioned solubility testing results also predicted that there should be inherent discrepancies in the employment scenarios of PAS-DPPZ-20, PAS-DPPZ-40, and PAS-DPPZ-60 versus PAS-DPPZ-80 and PAS-DPPZ. The molecular weights of these polymers were determined by high-temperature permeation gel chromatography (GPC). As the poor solubility of PAS-DPPZ-80 and PAS-DPPZ in DMF limited the tests, only data related to PAS-DPPZ-20, PAS-DPPZ-40, and PAS-DPPZ-60 are listed in Table 1. The combination of a high-number-average molecular weight (65.5–92.7 kDa) and a low degree in dispersion (1.5–2.3) set the foundation for the considerable photovoltaic characteristics [41].

### 2.4. Thermal Properties

The thermal properties of the polymeric materials were investigated via thermogravimetric analysis (TGA) and differential scanning calorimetry (DSC) (Table 1, Figures S3 and S4). The results indicated that the introduction of the phenazine group did not negatively affect the thermal stability in the system, and the polymer materials as a whole still maintained the advantageous thermal stability of PAS. All the polymers did not suffer any significant weight loss under the high-temperature test condition of 400 °C. Amongst them, the 5% thermal weight loss temperature (T_d5%_) of PAS-DPPZ-20 was as high as 512.0 °C in a nitrogen atmosphere. Furthermore, the glass transition temperature (T_g_) of the polymers progressed steadily with rising proportions on phenazine radicals in polymers, from 218.1 °C for PAS-DPPZ-20 to 275.5 °C for PAS-DPPZ. This result could be attributed to the fact that the presence of a planar structure with a strong rigidity underlying the phenazine matrix was able to restrict the movement of the polymer chain segments, leading to the PAS-DPPZ with the highest phenazine content exhibiting the minimum chain mobility [42]. Such an outstanding thermal stability undoubtedly broadens the scope of possibilities in which the material might be utilized and extends its service life.

**Table 1:** Molecular weights and thermal stabilities of PAS-DPPZs.

Polymer Code	GPC (kDa) ^a^			Thermal Stability (°C)	
	Mn	Mw	PDI	T_g_^b^	T_d5%_^c^
PAS-DPPZ-20	65.5	95.8	1.5	218.1	512.0
PAS-DPPZ-40	92.7	213.5	2.3	241.9	486.3
PAS-DPPZ-60	91.0	204.3	2.2	259.3	482.6
PAS-DPPZ-80	-	-	-	272.4	466.1
PAS-DPPZ	-	-	-	275.5	434.8

^a^ Mn, Mw, and PDI were determined by GPC in DMF and are reported relative to polystyrene standards. Molecular weight characteristics of PAS-DPPZ-80 and PAS-DPPZ could not be determined by GPC due to insolubility in DMF; ^b^ determined by DSC; ^c^ defined as the temperature at which 5% mass loss was observed as determined by TGA. Heating rate: 10 °C min^−1^, atmosphere: N_2_.

### 2.5. Optical and Electrochemical Properties

**Figure 1:**
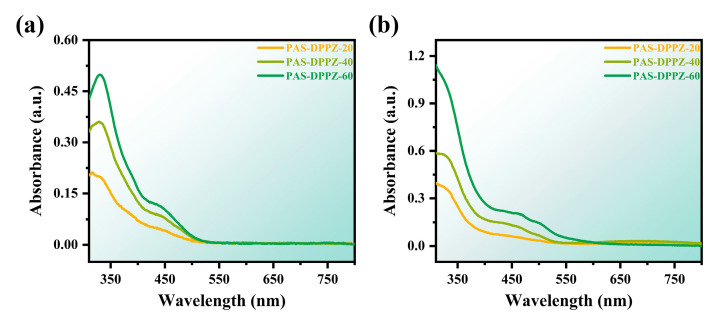
UV–Vis absorption spectra of PAS-DPPZ-20, PAS-DPPZ-40, and PAS-DPPZ-60 (**a**) for dilute solution in NMP and (**b**) for solid-state film.

The fundamental optical properties that characterize polymeric substances were explored with the help of UV–vis absorption spectroscopy. Limited by solubility, comparative trials were performed exclusively across PAS-DPPZ-20, PAS-DPPZ-40, and PAS-DPPZ-60. The data obtained are summarized in Table 2. The UV–Vis absorption spectra in NMP solution (Figure 1a) revealed that the intensity of the characteristic absorption peaks (316 nm–331 nm) ascribed to phenazine increased with the growing share of phenazine moieties as part of the polymer chain segments [43]. Concurrently, a new peak began to appear near the wavelength of 430 nm and continuously enhanced owing to the intramolecular charge transfer (ICT) effect that initiated between the electron-rich and electron-deficient groups. The spectral absorption for the solid-state film (Figure 1b) demonstrated a broadly comparable pattern of variation versus that of the solution. There was only a limited degree of redshift in the spectrum as a whole. The intensity of the redshift phenomenon was positively correlated with the total conjugation level within the polymer.

**Scheme 2:**
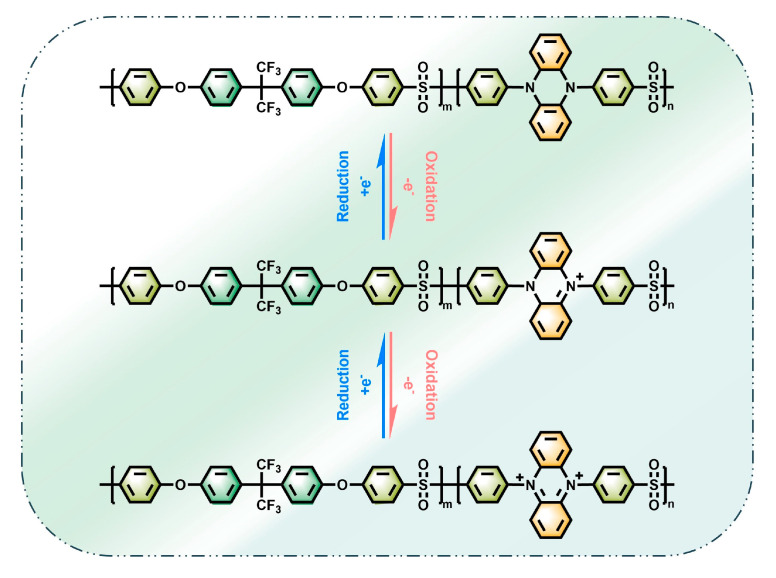
Specific redox process of PAS-DPPZs.

**Figure 2:**
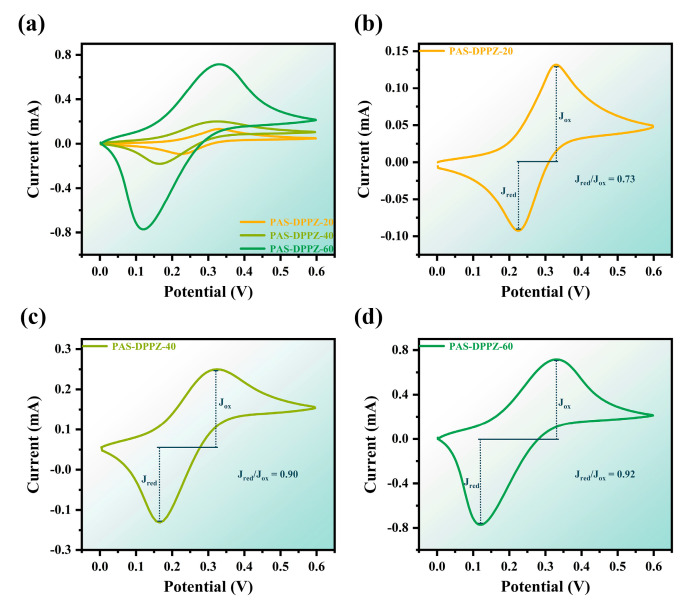
Cyclic voltammetric curves of comparison among PAS-DPPZ-20, PAS-DPPZ-40 and PAS-DPPZ-60 (**a**); PAS-DPPZ-20 (**b**); PAS-DPPZ-40 (**c**); and PAS-DPPZ-60 (**d**) recorded in 0.1 M TBAP/ACN (scanning rate: 0.1 V/s).

The cyclic voltammetry (CV) curves were measured in an inert gas atmosphere for the materials using indium tin oxide (ITO) glass spin-coated with a polymer film as the working electrode, a Ag/Ag^+^ electrode as the reference, and a platinum wire as the counter electrode (Figure 2 and Figure S5). The experiments were carried out in an acetonitrile (ACN) solution with tetrabutylammonium perchlorate (TBAP) at a concentration of 0.1 M. The specific redox process is illustrated in Scheme 2. With that in mind, the electrochemical properties of PAS-DPPZ-20, PAS-DPPZ-40, and PAS-DPPZ-60 were evaluated. Primarily, a pair of redox peaks existed in the cyclic voltammetry curves of all three polymers, which corresponded to the reversible redox process of phenazine [44]. Moreover, increasing the proportion of phenazine groups in the polymers contributed to the overall electrochemical enhancement upon comparison. It was specifically reflected by the growth in the redox peak current density in the cyclic voltammetry curves; the reduction in the onset oxidation potential (E_onset_) and half-wave potential ($E_{\mathrm{ox}1/2}$) (E_onset_: 0.18 V–0.11 V, E: 0.26 V–0.20 V); the increase in the ratio between the reduction and oxidation peak current (J_red_/J_ox_) (0.73–0.92); and the decrease in electrochemical degradation after 10,000 consecutive cyclic voltammetry cycles. In the CV curve, the higher peak current represented the better electrochemical activation of the material; the closer the value of J_red_/J_ox_ to one, the greater the reversible extent of this electrochemical redox process [45]; and lower E_onset_ and $E_{\mathrm{ox}1/2}$ commonly imply more excellent electrochemical stability. The significant improvement in these indicators was undoubtedly evidence of the success of the modification strategy that introduced phenazine-based redox-active centers into high-performance polymers in order to endow them with electrochemical properties. This initiative made the already superior physicochemical properties of PAS break through the constraints imposed by their own lack of electroactivity in order to shine in electrochemical-related areas. This tremendously expands usage opportunities and enhances the actual application value of the materials.

The introduction of phenazine groups bringing about an enhancement in electrochemical properties could also be explained from the viewpoint of molecular orbitals and energy band gaps (E_g_). The highest occupied molecular orbital (HOMO) energy levels of PAS-DPPZ-20, PAS-DPPZ-40, and PAS-DPPZ-60 were calculated based on the onset oxidation potentials determined by cyclic voltammetry, with ferrocene as the internal standard, as tabulated in Table 3. The E_g_ and lowest unoccupied molecular orbital (LUMO) energy levels of the three polymers were summarized with the assistance of the onset absorption wavelength (λ_onset_) obtained from UV–Vis absorption spectra and the relevant formulae. The data revealed that the strengthened D–A (donor–acceptor) effect brought about by the elevated content of phenazine moieties was reflected at the molecular level by a decrease in E_g_ (2.33 eV–2.19 eV). That finding coincided with the results concerning the gradual progression of the long-term stability tests in the CV curves.

**Table 2:** Optical characteristics of PAS-DPPZ-20, PAS-DPPZ-40, and PAS-DPPZ-60.

Polymer Code	Solution	Film	
	λ_max_ (nm)	λ_max_ (nm)	λ_onset_ (nm)
PAS-DPPZ-20	316,434	445	533
PAS-DPPZ-40	328,437	452	546
PAS-DPPZ-60	331,439	460	565

**Table 3:** Electrochemical properties, energy levels, and energy band gaps of PAS-DPPZ-20, PAS-DPPZ-40, and PAS-DPPZ-60.

Polymer Code	Oxidation ^a^ (V)		E_g_^b^ (eV)	Energy Level ^c^ (eV)	
	E_onset_	E_ox1/2_		HOMO	LUMO
PAS-DPPZ-20	0.18	0.26	2.33	−4.98	−2.65
PAS-DPPZ-40	0.13	0.21	2.27	−4.93	−2.66
PAS-DPPZ-60	0.11	0.20	2.19	−4.91	−2.72

^a^ Obtained from CV curve; ^b^ E_g_ = 1240/λ_onset_. ^c^ LUMO = HOMO-E_g_.

### 2.6. Spectroelectrochemistry and Electrochromic Properties

**Figure 3:**
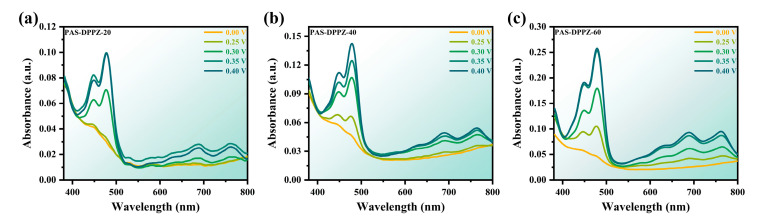
Spectroelectrochemistry of PAS-DPPZ-20 (**a**), PAS-DPPZ-40 (**b**), and PAS-DPPZ-60 (**c**) under the same experimental conditions.

Encouraged by the promising electrochemical long-term stability in cyclic voltammetry testing, spectroelectrochemical measurements were performed to evaluate the electrochromic potency of PAS-DPPZ-20, PAS-DPPZ-40, and PAS-DPPZ-60. Spectroelectrochemical testing is one of the most common means to confirm the presence or absence of electrochromic properties through the combination of electrochemical workstation and UV–Vis absorption spectroscopy to measure the spectral absorption change in a material in situ under different applied voltages. The concrete experimental conditions were analogous to those of cyclic voltammetry tests. The variation in the UV–Vis absorption spectra of the three materials to be assessed with the applied voltage are illustrated in Figure 3. The patterns of the three spectral lines were highly consistent, with a new absorption band appearing at 400 nm–500 nm in the process of voltage gradient elevation. In parallel, moderate-intensity broadband absorption was observed in the visible and near-infrared regions from 600 nm to 800 nm, where the highest intensity peaks were located at 628 nm, 688 nm, and 763 nm. When the voltage rose to 0.4 V, that round of absorption enhancement reached saturation, suggesting that the stage-by-stage oxidation process of the electroactive units in the polymer was essentially complete. The polymer film on the working electrode surface at this point turned from the initial light yellow to the green color of the radical cation [46,47,48]. Additionally, further comparisons revealed that the identical voltage alterations did not result in the same modification of the spectral absorption intensities for the three polymers. The larger the proportion of phenazine groups, the more the spectral intensity changed under consistent conditions, which implied that the observed color variation was more pronounced. As a result, when it comes to the practical implementation of electrochromism, it was predicted that PAS-DPPZ-60 is more competitive than PAS-DPPZ-20 and PAS-DPPZ-40.

**Figure 4:**
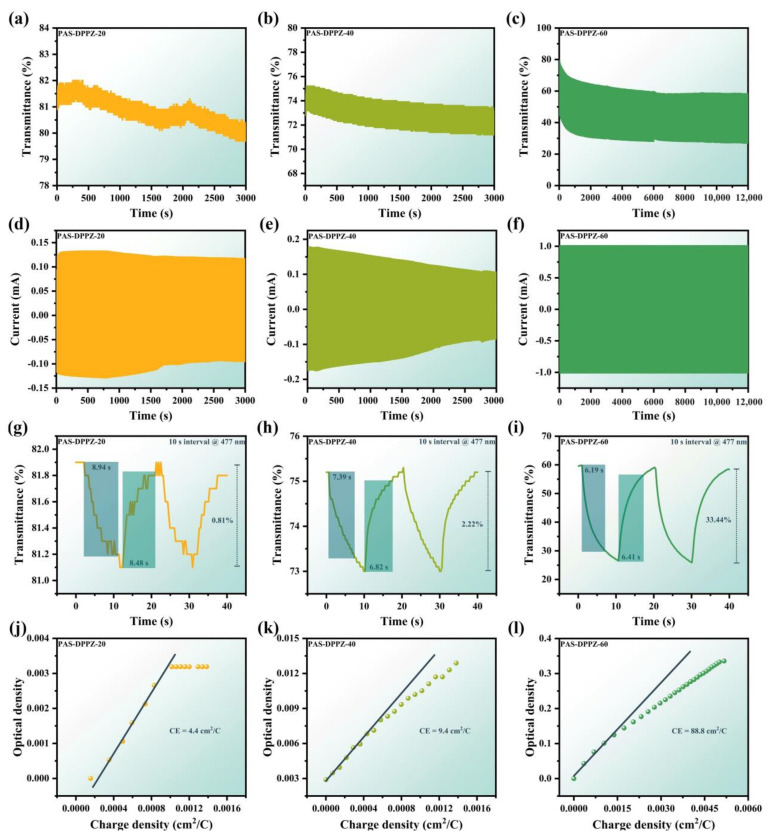
(**a**–**c**) Variation in transmittance in electrochromic stability tests for PAS-DPPZ-20, PAS-DPPZ-40, and PAS-DPPZ-60; (**d**–**f**) variation in current at the working electrode in electrochromic stability tests for PAS-DPPZ-20, PAS-DPPZ-40, and PAS-DPPZ-60; (**g**–**i**) coloring/bleaching time of PAS-DPPZ-20, PAS-DPPZ-40, and PAS-DPPZ-60; (**j**–**l**) coloration efficiency of PAS-DPPZ-20, PAS-DPPZ-40, and PAS-DPPZ-60 (obtained by linear fitting).

Tangible electrochromic performance metrics were derived by monitoring the transmittance of electrochromic materials at specific wavelengths with applied pulses by the chronoamperometry approach. The duration to accomplish 90% of the total transmittance change is commonly defined as the electrochromic switching time required for the material [49]. Under identical test conditions (pulse voltage and time interval), as illustrated in Figure 4, the transmittance evolution of the polymers enhanced dramatically as the proportion of phenazine within the system increased from 0.81% in PAS-DPPZ-20 to 33.44% in PAS-DPPZ-60. The electrochromic switching time was further shortened from nearly 9 s (coloring time: 8.94 s, bleaching time: 8.48 s) for PAS-DPPZ-20 to just about 6 s (coloring time: 6.19 s, bleaching time: 6.41 s) for PAS-DPPZ-60. All these performance improvements could be traced to the electroactive enhancement brought to the material by the increase in phenazine occupancy.

The long-term service reliability of electrochromic behavior is an alternative indicator that called for attention during actual practice. In this regard, PAS-DPPZ-20, PAS-DPPZ-40, and PAS-DPPZ-60 were tested for electrochromic cycling stability. The cyclic stability of PAS-DPPZ-20 with the lowest phenazine content was found to be the worst, followed by PAS-DPPZ-40 with the medium phenazine content, while PAS-DPPZ-60 with the highest phenazine content exhibited the best cyclic stability among the three. During an uninterrupted electrochromic switching cycle of up to 12,000 s (600 cycles), there was no significant degradation in either the transmittance change or the corresponding current response. The origin of its excellent electrochromic cycling stability is the highly reversible nature of the electrochemical reaction, with the result that the charge injection and extraction capabilities of the electrochemical redox process are relatively well-matched. To be specific, the charge injection and extraction capabilities could be predicted by performing cyclic voltammetry tests on the materials to be tested. The oxidation peak current density (J_ox_) and reduction peak current density (J_red_) in the curves represented, to some extent, the charge injection and extraction capability during the electrochemical redox process [38]. Regarding the series of materials structurally similar to each other, the closer the J_red_/J_ox_ value is to one, the higher the degree of matching and the higher the cyclic stability. The experimentally calculated J_red_/J_ox_ values of PAS-DPPZ-20, PAS-DPPZ-40, and PAS-DPPZ-60 consecutively enlarged up to 0.73, 0.90, and 0.92, respectively, which exactly corresponded to the significantly enhanced cycling stability results. Moreover, the fitting outcome of the coloration efficiency (CE) reconfirmed the considerable contribution of the elevated phenazine ratio to the upgrade in the electrochemical performance of the system. With a linear fit of the charge density and optical density relationship curve, the slope yielded is, as defined, the coloration efficiency [50]. The magnitude of the value is an indication of how much electrical energy the material is capable of converting when undergoing the electrochromic process. As is obvious, materials with large coloration efficiencies consumed less energy during the electrochromic process and thus offered a higher utility value. When the proportion of phenazine groups increased from 20% to 60%, the coloration efficiency in the system achieved an order of magnitude upgrade from 4.4 cm^2^/C to 88.8 cm^2^/C. Comparing the electrochromic performance indices, it was not surprising to notice that the comprehensive performance of PAS-DPPZ-60, which possessed a high proportion of phenazine, was much better than that of PAS-DPPZ-20 and PAS-DPPZ-40. Together with the introduction of phenazine into the poly(aryl ether sulfone) system, it demonstrated another way to achieve the purpose of imparting stable electrochemical activity to the PAS system without damaging its original benefits.

### 2.7. Battery Properties

**Figure 5:**
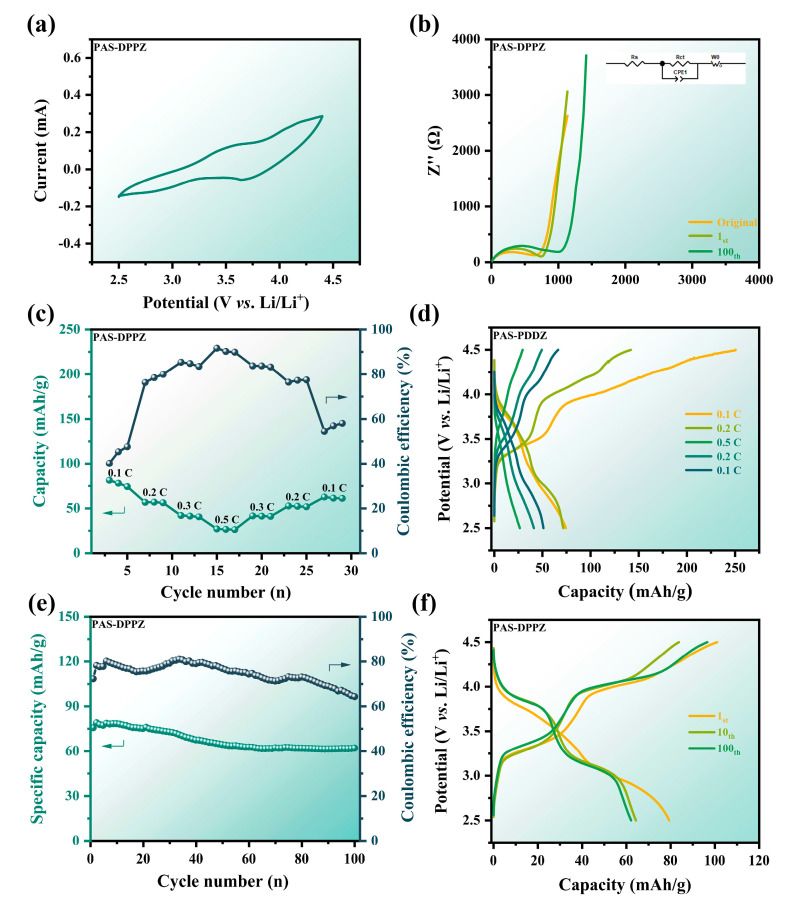
The batteries of PAS-DPPZ with (**a**) CV curves (1.5–3.5 V) at 0.1 mV/s, (**b**) Nyquist plots (initial, 1, and 100 cycles), and corresponding equivalent circuit diagrams, (**c**,**d**) the rate capability and charge/discharge curves at 0.1 C, 0.2 C, 0.3 C and 0.5 C, (**e**) cycling performance and coulombic efficiency at 0.1 C, and (**f**) charge/discharge curves (1, 10, and 100 cycles) at 0.1 C.

Whether PAS-DPPZ, which has pretty poor solubility in common organic solvents, could be utilized as an organic cathode material was tested using a coin battery. The detailed assembly steps are shown in the Supporting Information. In order to investigate the redox potential of the phenazine derivative electrode, the battery of PAS-DPPZ was subjected to cyclic voltammetry tests at a scan rate of 0.1 mV/s (2.5–4.4 V). As shown in Figure 5a, the polymer PAS-DPPZ exhibits two pairs of redox peaks corresponding to two consecutive single-electron-transfer redox reaction processes. Among them, the two pairs of reduction peak potentials of PAS-DPPZ were about 3.0 V and 3.8 V (vs. Li/Li^+^), and the oxidation peak potentials were about 3.5 V and 4.1 V (vs. Li/Li^+^), respectively. Figure 5b shows the Nyquist plots of PAS-DPPZ at different cycles. In particular, the initial impedance of PAS-DPPZ was the smallest and increases with the number of cycles. After 100 cycles, the impedance increased to 1046 Ω. In addition, in order to further verify that the PAS-DPPZ material has the potential to be used as a cathode material, the battery of PAS-DPPZ was subjected to charge/discharge tests at different current densities and constant current charge/discharge, respectively. The test results of PAS-DPPZ at 0.1 C, 0.2 C, 0.3 C, and 0.5 C multiplicity are shown in Figure 5c. Among them, the discharge specific capacity at 0.1 C was about 82 mAh/g, and the capacity was about 27 mAh/g when the current density was increased to 0.5 C, with a capacity retention rate of 33%. Figure 5d shows the charge–discharge curves of PAS-DPPZ at 0.1 C, 0.2 C, and 0.5 C, where the different curves exhibit two obvious charge–discharge plateaus, while the voltage difference between the two plateaus is about 0.8 V, which is consistent with the results of the CV test above. The above results indicate that PAS-DPPZ containing an active center can significantly exhibit cycling stability when used as an electrode material after being used in a phenazine derivative as a polymer linking unit. Furthermore, it can be observed from Figure 5e that the first discharge specific capacity of the electrode PAS-DPPZ was about 76 mAh/g, and the capacity was maintained at about 62 mAh/g after 100 cycles, which was about 82% of the initial capacity. Additionally, it can be observed in Figure 5f that the discharge curves of the 10th and 100th cycles almost coincide with the discharge curve of the first cycle, which further demonstrates the good cycling stability of PAS-DPPZ.

In summary, the test results demonstrate that simply adjusting the occupancy rate of phenazine could realize a leap in the field of material applications, which reflects the ingenious and practical design concept.

## 3. Materials and Methods

### 3.1. Materials

All reagents were received without any further purification. Phenazine, sodium dithionite, and bisphenol AF were obtained from Energy Chemical (Shanghai, China). Anhydrous ethanol (EtOH), N-methyl-2-pyrrolidone (NMP), and potassium carbonate (K_2_CO_3_) were sourced from China National Medicines Corporation Ltd. (Beijing, China). K_2_CO_3_ had to be ground and vacuum-dried before use. Tetrabutylammonium perchlorate (TBAP) and ultra-dry-grade acetonitrile (ACN) were purchased from Macklin (Shanghai, China). The materials required for assembling the button cell were commercially available. The ITO glass was sonicated sequentially with deionized water, acetone, and ethanol for 15 min before use.

### 3.2. Methods

The polymer structures were determined by nuclear magnetic resonance hydrogen (^1^HNMR) spectroscopy at 300 MHz (Bruker Avance, Bruker BioSpin, Rheinstetten, Germany) and Fourier transform infrared (FT-IR) spectroscopy (Nicolet AVATAR 360). The solvent for ^1^HNMR was deuterated trichloromethane (CDCl_3_), and the blank background for FT-IR was potassium bromide (KBr). The eluent for gel permeation chromatography (GPC, Agilent, Palo Alto, CA, USA) to measure the molecular weight of the polymers was N,N-dimethylformamide (DMF). The standard sample was monodisperse polystyrene. Thermogravimetric analysis (TGA) was performed with a PerkinElmer Pyris 1 TGA instrument (PerkinElmer, MA, USA) and differential scanning calorimetry (DSC) with a DSC 821e apparatus (Mettler, Zurich, Switzerland). The gas atmosphere for both was nitrogen, and the heating rate was 10 °C min^−1^. Electrochemical correlation data were obtained with the classical three-electrode system and evaluated using a CHI660D electrochemical workstation (Chenhua, Shanghai, China). Optical properties were assessed on a UV-1600 spectrophotometer (Macy, Shanghai, China). The measurement of the button cell was completed in a NEWARE battery test system.

## 4. Conclusions

A functionalized synthetic strategy for introducing phenazine-based redox centers into high-performance polymer systems was proposed. A family of PAS-DPPZs with electrochemical activeness and long-term stability was prepared through a facile C–N coupling method. The system exhibits distinct electrochemical features and suitable application domains depending on the proportion of phenazine groups. Among them, PAS-DPPZ-60 stands out for its electrochromic performance in comparison to all other polymers. There was almost no performance degradation during the long-time cycling test of 12,000 s. Meanwhile, PAS-DPPZ, with the largest percentage of phenazine, performed well as an organic battery cathode material. With the assistance of phenazine to strengthen the free domain and stabilize the free radicals within the system, batteries fabricated from PAS-DPPZ not only possess a theoretical capacity of 126 mAh g^−1^ but also show a low capacity decay of less than 20% over 100 charging/discharging cycles. Such a functionalization strategy, incorporating several benefits of PAS and phenazine, not only expands the range of high-performance polymers and the scope of their implementations but also offers concepts for future material modification.

## Data Availability

Data are contained within the article.

## Supplementary Materials

The following supporting information can be downloaded at: https://www.mdpi.com/article/10.3390/molecules29071618/s1. Figure S1: NMR spectra of PAS-DPPZs; Figure S2: FT-IR spectra of PAS-DPPZs; Figure S3: TGA curve of PAS-DPPZs; Figure S4: DSC curve of PAS-DPPZs; Figure S5: Comparison diagram of 10,000-cycle voltammetry curves of PAS-DPPZ-20 (a); PAS-DPPZ-40 (b) and PAS-DPPZ-60 (c); Table S1: Table of PAS-DPPZs solubility test results; and procedure of CR2023 coin battery assembly.
