# Supplementary material for: Synthesis and Characterization of Phenazine-Based Redox Center for High-Performance Polymer Poly(aryl ether sulfone)-5,10-Diphenyl-dihydrophenazine
Source: Molecules. 2024 Apr 3;29(7):1618. doi: 10.3390/molecules29071618 (PMC11013081; doi:10.3390/molecules29071618)
Supplement: Supplementary file 1 [file molecules-29-01618-s001.zip › molecules-2927384-supplementary.pdf]

Synthesis and Characterization of Phenazine-Based Redox Center for High-Performance Polymer Poly(Aryl Ether Sulfone)-5, 10-Diphenyl-Dihydrophenazine

Qilin Wang 1, Xuehan Wang 1, Yuehui Zhai 2, Zhibo Zheng 3, Huilin Shen 1, Yuntao Han 1,*, Zheng Chen 1,* and Zhenhua Jiang 1

1 Engineering Research Center of Special Engineering Plastics, Ministry of Education, National and Local Joint Engineering Laboratory for Synthetic Technology of High Performance Polymer, College of Chemistry, Jilin University, Qianjin Street 2699, Changchun 130012, China; wangql22@mails.jlu.edu.cn (Q.W.); xuehan23@mails.jlu.edu.cn (X.W.); shenhl22@mails.jlu.edu.cn (H.S.); jiangzhenhua@jlu.edu.cn (Z.J.)
2 School of Materials and Energy, University of Electronic Science and Technology of China, Chengdu 611731, China; zhaiyuehui@uestc.edu.cn (Y.Z.)
3 Department of Chemical Engineering and Applied Chemistry, College of Chemistry, Jilin University, Qianjin Street 2699, Changchun 130012, China; zhengzb22@mails.jlu.edu.cn
* Correspondence: hanyuntao@boe.com.cn (Y.H.); chenzheng2013@jlu.edu.cn (Z.C.)

Table of Contents

1. NMR spectra of PAS-DPPZs...3

2. FT-IR spectra of PAS-DPPZs...4

3. TGA curve of PAS-DPPZs...5

4. DSC curve of PAS-DPPZs 6

5. Comparison diagram of 10,000-cycle voltammetry curves of PAS-DPPZ-20 (a); PAS-DPPZ-40 (b) and PAS-DPPZ-60 (c)...7

6. Table of PAS-DPPZs solubility test results...8

7. Procedure of CR2023 coin battery assembly...9

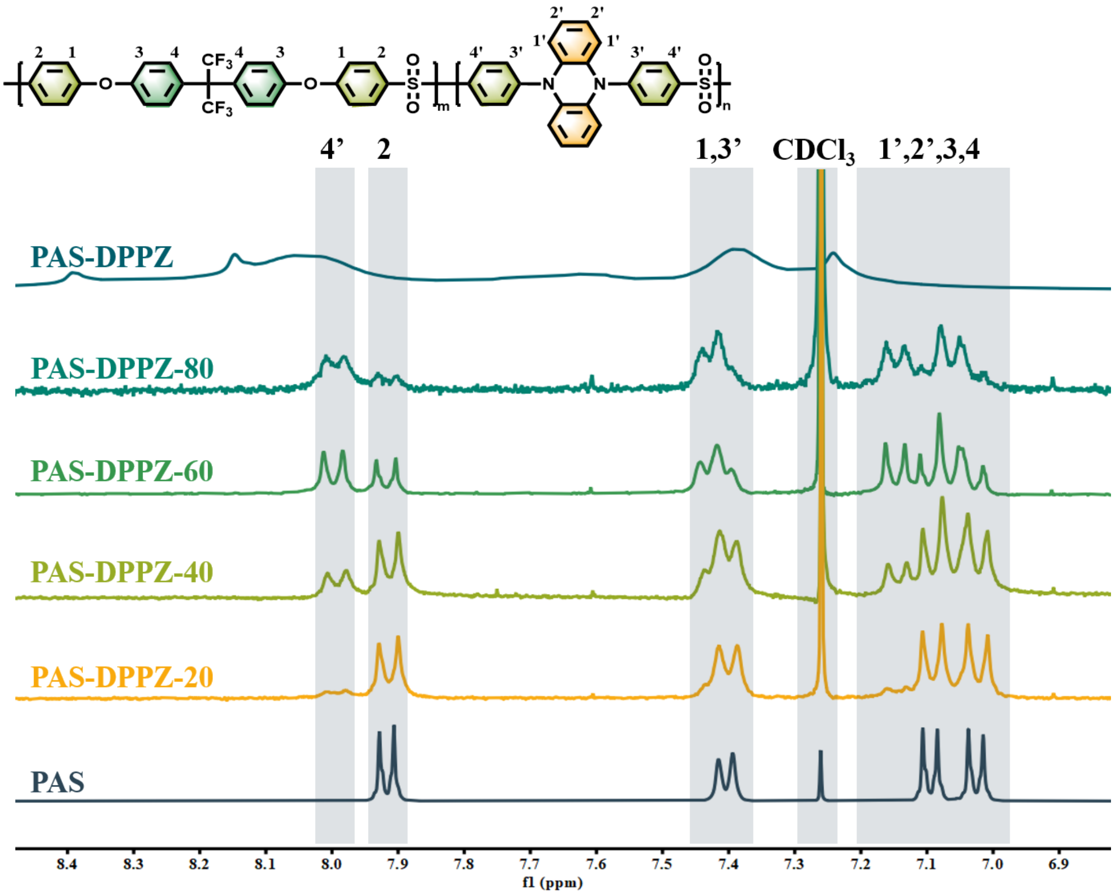

Figure S1. ^{1}HNMR spectra of PAS-DPPZs (Due to solubility limitations, the NMR tests for PAS-DPPZs were performed in TFA solvents, while the other polymers were in chloroform.).

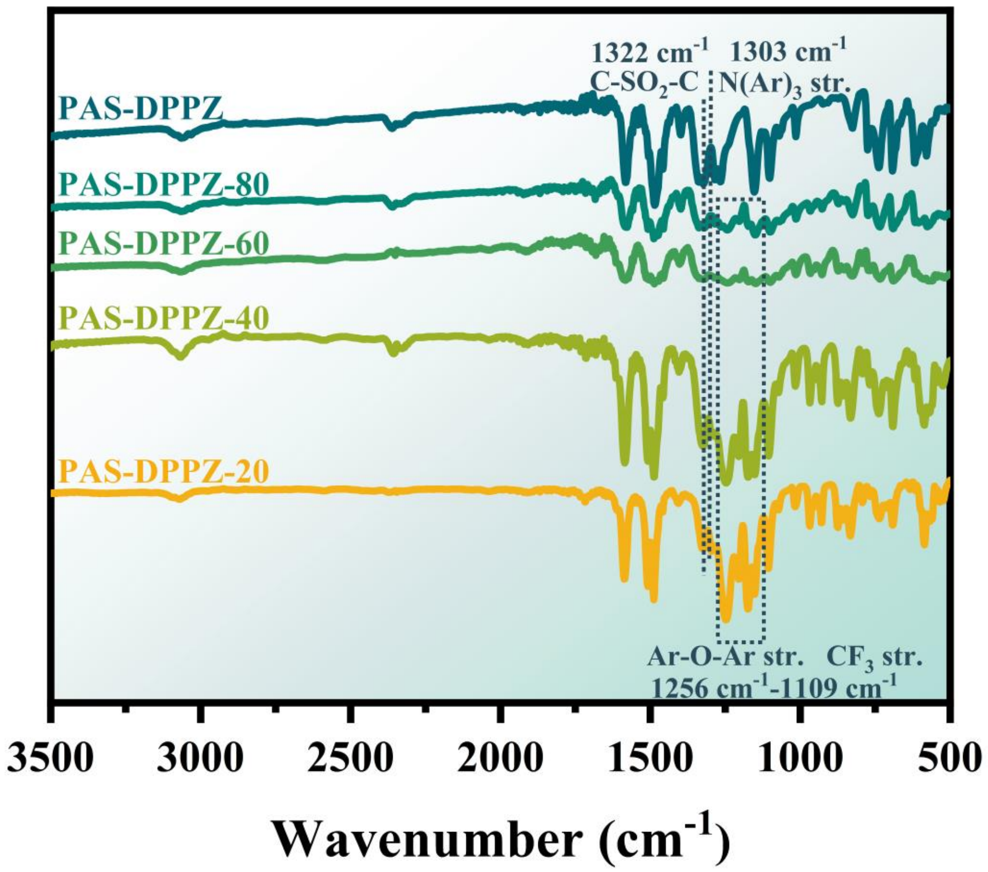

Figure S2. FT-IR spectra of PAS-DPPZs.

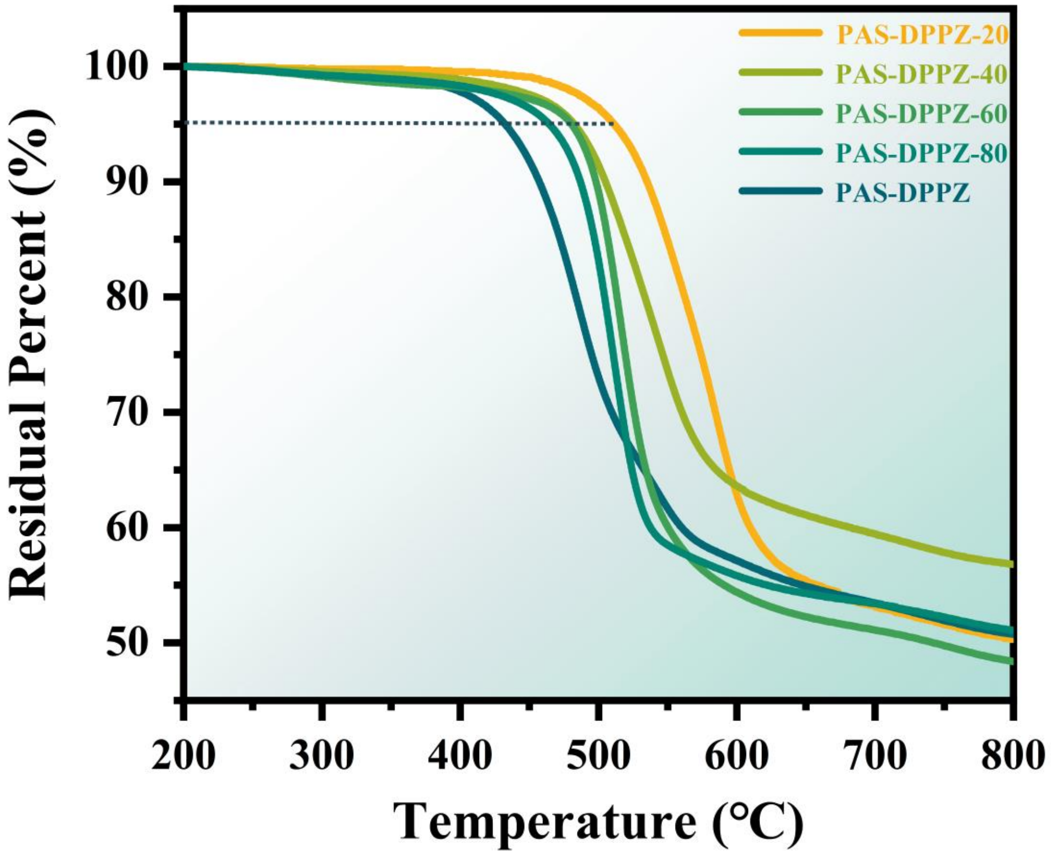

Figure S3. TGA curve of PAS-DPPZs.

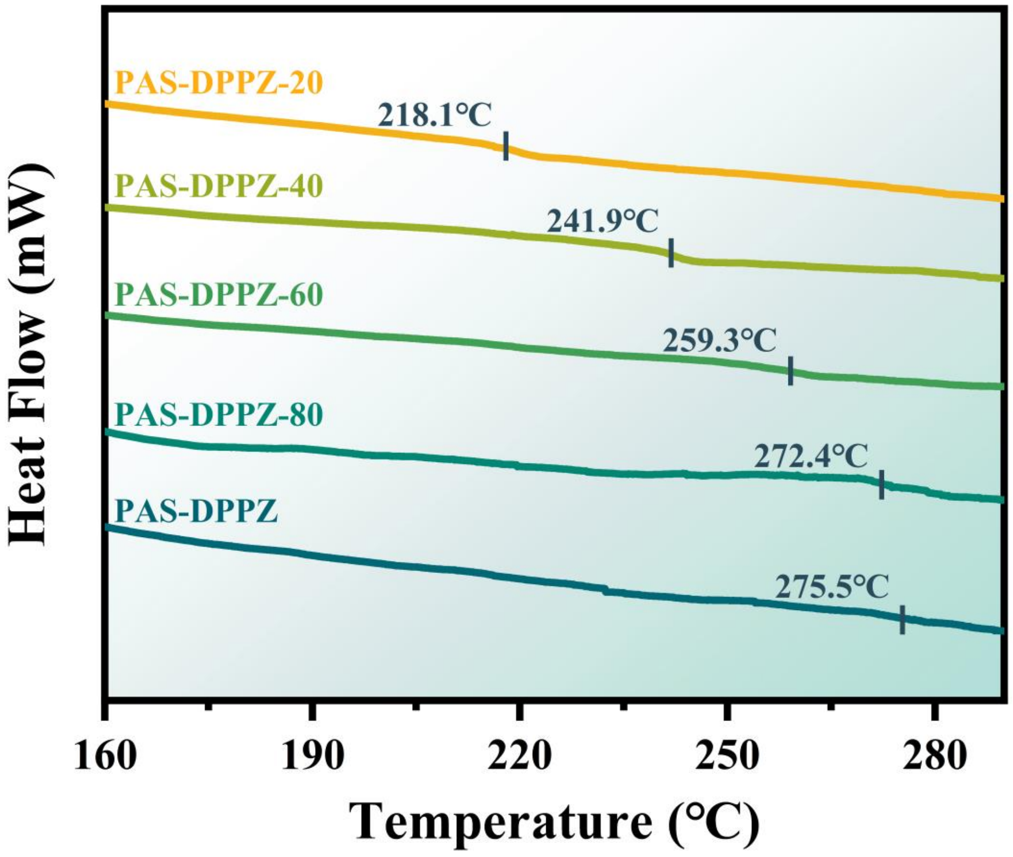

Figure S4. DSC curve of PAS-DPPZs.

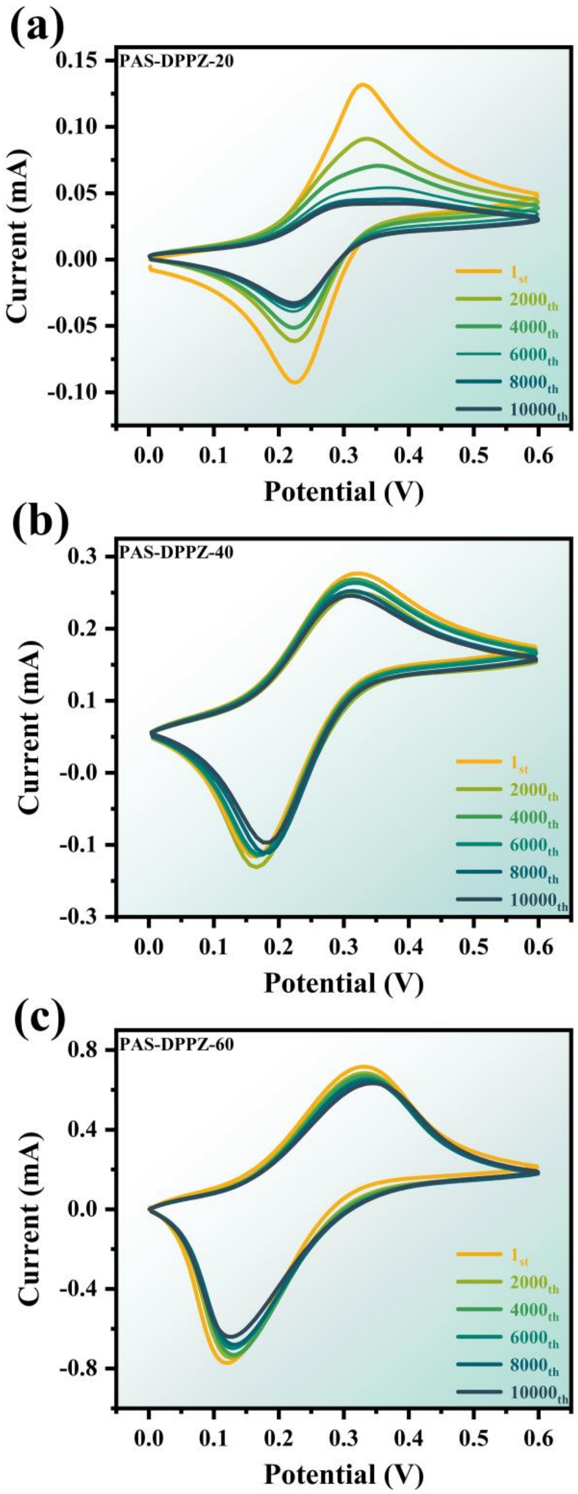

Figure S5. Comparison diagram of 10,000-cycle voltammetry curves of PAS-DPPZ-20 (a); PAS-DPPZ-40 (b) and PAS-DPPZ-60 (c) in 0.1 M TBAP/ACN solution at 0.1 V/s scanning rate.

Table S1. Solubility test results of PAS-DPPZs

Polymer Code	Solvent[a]							
	THF	CH_2Cl_2	$CHCl_3$	DMF	DMAc	DMSO	NMP	ACN
PAS-DPPZ-20	++	++	++	++	++	+	++	-
PAS-DPPZ-40	++	+	++	++	++	+	++	-
PAS-DPPZ-60	-	-	++	+	++	-	++	-
PAS-DPPZ-80	-	-	-	-	-	-	-	-
PAS-DPPZ	-	-	-	-	-	-	-	-

++: Soluble at room temperature; +: soluble on heating; -: insoluble on heating.

a) Qualitative solubility was determined with as 10 mg of polymer in 1 mL of solvent.

Procedure of CR2023 coin battery assembly

PAS-DPPZ, conductive carbon black (Super P) and polyvinylidene fluoride (PVDF) were dissolved in N-methyl-2-pyrrolidone after grinding for 1h in a weight ratio of 7:2:1. It was dried at 80°C for 12 h after being uniformly coated on aluminum foil. After that, it was cut into electrode sheets of 12 mm diameter and weighed. The average loading of the electrode active substance was about 0.5 mg/cm. Subsequently, it was transferred to a glove box (H_2O < 1 ppm, O_2 < 1 ppm) for assembly and packaging. In particular, the electrode, diaphragm (Celgard 2500), electrolyte (1 M $LiPF_6$ in EC/DEC, v/v = 1:1), and lithium metal sheet anode (0.45 mm thickness, 15.8 mm diameter) were assembled and sealed using CR2032 coin battery.
